# New additions to *Omphalotaceae* (*Agaricales*, *Basidiomycota*): two new rhizomorph-forming species from China

**DOI:** 10.3897/mycokeys.131.184214

**Published:** 2026-04-09

**Authors:** Ji Peng Li, Jun Gao, Yi Li, Meng Le Xie, Chang Tian Li

**Affiliations:** 1 Engineering Research Center of Edible and Medicinal Fungi, Ministry of Education, Jilin Agricultural University, Changchun 130118, China School of Food Science and Engineering, Yangzhou University Yangzhou China; 2 Nanjing Institute of Environmental Sciences, Ministry of Ecology and Environment, Nanjing 210042, China Engineering Research Center of Edible and Medicinal Fungi, Ministry of Education, Jilin Agricultural University Changchun China; 3 School of Food Science and Engineering, Yangzhou University, Yangzhou 225127, China Nanjing Institute of Environmental Sciences, Ministry of Ecology and Environment Nanjing China

**Keywords:** *Gymnopus sect. Androsacei*, phylogeny, *
Pseudomarasmius
*, taxonomy

## Abstract

Two new rhizomorph-forming species within the family *Omphalotaceae*, *Gymnopus fuscostipes***sp. nov**. and *Pseudomarasmius brunneodiscus***sp. nov**., are proposed based on morpho-molecular evidence. Both species are characterized by marasmioid basidiomata, brownish pileus, and black rhizomorphs. Detailed morphological descriptions, color photos, and illustrations are presented herein, as well as comparisons with closely related species.

## Introduction

Rhizomorphs are hair- or wire-like macrostructures that have evolved multiple times independently and are primarily distributed in at least six genera of the *Omphalotaceae*, including *Collybiopsis* (J. Schröt.) Earle, *Gymnopus* (Pers.) Gray, *Paramycetinis* R.H. Petersen, *Pseudomarasmius* R.H. Petersen & K.W. Hughes, *Pusillomyces* J.S. Oliveira, and *Paragymnopus* J.S. Oliveira (Antonín and Noordeloos 2010; Petersen and Hughes 2016, 2020; Desjardin and Perry 2017; Li et al. 2024; Oliveira et al. 2024). Among these taxa, *Pseudomarasmius* R.H. Petersen & K.W. Hughes and *G. sect. Androsacei* (Kühner) Antonín & Noordel. have long been closely linked both morphologically and taxonomically. Both share marasmioid basidiomata, rhizomorphs, and a pileipellis composed of diverticulate hyphae (Antonín and Noordeloos 2010; Petersen and Hughes 2020). The genus *Pseudomarasmius* has recently been proposed to accommodate species lacking or nearly lacking clamp connections that were previously placed in *Marasmius* Fr. and *Gymnopus sect. Androsacei* (Petersen and Hughes 2020). Gilliam (1975) described *Marasmius pallidocephalus* within *Ma. sect. Androsacei* Kühner only reported clamp connections in the pileus trama. However, Desjardin (1989) subsequently noted that clamp connections are absent in all tissues of this species. This morphological discrepancy complicated the generic placement of the taxon.

While morphological interpretations varied, molecular phylogenetic analyses provided new insights that triggered a series of taxonomic revisions. With phylogenetic analyses revealing the type species of *Ma. sect. Androsacei* within the *Gymnopus* clade, the section was transferred to that genus (Mata et al. 2004; Wilson and Desjardin 2005; Noordeloos and Antonín 2008). However, the LSU-based phylogeny in Petersen and Hughes (2016) recovered *Ma. pallidocephalus* as a separate lineage close to *Rhodocollybia* Singer and *Connopus* R.H. Petersen, distant from *G. sect. Androsacei*. Subsequently, Petersen and Hughes (2017) and Oliveira et al. (2019) indicated the possibility that this species does not fit within any known genus. Petersen and Hughes (2020) then examined the type specimen of *Ma. pallidocephalus* and proposed the genus *Pseudomarasmius* to accommodate it as the type species, after confirming the absence of clamp connections.

Despite the clear phylogenetic distinction between *Pseudomarasmius* and *G. sect. Androsacei*, species in these two groups often share similar morphological features, which complicates their identification in the field. During a recent survey of macrofungi in southern China, several rhizomorph-forming specimens were collected. Morphological examination combined with molecular phylogenetic analyses revealed that these collections may represent two undescribed species, i.e., one in *G. sect. Androsacei* and one in the genus *Pseudomarasmius*. Color photos of basidiomata, illustrations of microscopic structures, comparisons with related species, and phylogenetic trees were provided.

## Material and methods

### Abbreviations

The abbreviations used for genus names are *Connopus* = *C.*, *Gymnopus* = *G.*, *Marasmius* = *Ma.*, *Micromphale* = *Mi.*, *Paragymnopus* = *Pg.*, and *Pseudomarasmius* = *Ps.* The abbreviations used in phylogeny are Akaike information criterion with small-sample correction = **AICc**, average standard deviation of split frequencies = **ASDSF**, Bayesian inference = **BI**, Bayesian information criterion = **BIC**, bootstrap values = **BS**, internal transcribed spacer = **ITS**, effective sample size = **ESS**, large subunit ribosomal RNA gene = **LSU**, maximum likelihood = **ML**, Markov chain Monte Carlo = **MCMC**, posterior probability = **PP**, and 5.8S ribosomal RNA gene = **5.8S**.

### Morphology

Macroscopic characters were recorded from fresh basidiomata in the field and later checked against photographs. Color descriptions followed the alphanumeric codes and associated color terms of Kornerup and Wanscher (1978). The number of full-length lamellae is indicated by ‘L,’ and ‘l’ represents the number of lamellulae tiers. For microscopic examination, hand-cut sections were rehydrated in 5% KOH; Congo Red was used to stain hyaline elements, and Melzer’s reagent was applied to test for amyloid or dextrinoid reactions. Microscopic structures were observed using a ZEISS Axioscope 5 microscope, and measurements were obtained using ZEN 3.1 software. Values initially recorded to three decimal places were rounded to a single decimal place for analysis. The notation [*n* = *x*, *m* = *y*] indicates that x structures were measured from y basidiomata. Basidiospore dimensions (length × width) are given as (a)b–c(d), where b–c covers 90% of the observed range, and a and d represent the extreme values. The spore length/width ratio is expressed as Q, and Qm denotes the mean Q. Mean basidiospore length, mean width, and Qm are additionally presented with standard deviations. Measurements refer to the main body of each structure, excluding appendages such as apiculi and sterigmata.

### Molecular

Total genomic DNA was extracted from dried specimens using the NuClean Plant Genomic DNA Kit (Cowin Biotech Co., Ltd., Taizhou, China). The nuclear ribosomal large subunit (nrLSU) and internal transcribed spacer (ITS) were amplified using primer pairs LR0R/LR5 and ITS5/ITS4 (ITS4B), respectively (White et al. 1990; Vilgalys and Hester 1990; Cubeta et al. 1991; Gardes and Bruns 1993). PCR products were generated according to the procedure of Li et al. (2024) and sent to Sangon Biotech (Shanghai, China) for Sanger dideoxy sequencing. Direct Sanger sequencing failed for HMJAU 60404 and HMJAU 60453 due to polymorphism, and the amplicons were then subjected to TA cloning and sequencing.

### Phylogeny

Phylogenetic analyses were conducted using a dataset concatenating ITS and LSU sequences. Based on the phylogenetic relationships reconstructed by Li et al. (2024), two datasets were analyzed in this study: Dataset 1 for the phylogenetic analysis of *Gymnopus*, using two *Paragymnopus* species as outgroups, and Dataset 2 for the phylogenetic analysis of *Pseudomarasmius*, using *Connopus acervatus* (Fr.) K.W. Hughes, Mather & R.H. Petersen as an outgroup. Sequences used in this study are listed in Table 1. Multiple sequence alignments were generated using MAFFT v7.526 (Katoh and Standley 2013). The L-INS-i algorithm was employed to maximize accuracy, and gappy regions were preserved to maintain the integrity of variable regions. The alignment was carried out in two steps. An initial alignment was produced to assess sequence quality, during which terminal regions showing irregular variation within otherwise conserved blocks were removed. After this trimming, gap characters were deleted, and the cleaned sequences were re-aligned using the same MAFFT settings. Model selection was performed in IQ-TREE v2.4.0, allowing for partition merging to optimize model fit (Minh et al. 2020). The BIC and AICc criteria were used to select the model schemes for maximum likelihood and Bayesian analyses, respectively. Maximum-likelihood inference was performed in IQ-TREE v2.4.0 with 5000 ultrafast bootstrap replicates (Minh et al. 2020). The best-scoring maximum-likelihood (ML) tree was retained as the final topology. Bayesian inference was performed in MrBayes 3.2.7a as an additional analysis to assess branch support and congruence with the ML topology, with two independent runs of four chains, allowing a maximum of 10 million generations and sampling every 500 generations (Ronquist et al. 2012). The analyses were automatically terminated once the average standard deviation of split frequencies fell below 0.01. MCMC performance was evaluated in Tracer v1.7 by inspecting LnL trace plots (Rambaut et al. 2018). Posterior probabilities were subsequently calculated by mapping the posterior tree samples onto the ML topology using SumTrees v5.0.8 (Moreno et al. 2024). Bayesian posterior probabilities (PP) ≥ 0.95 and maximum-likelihood ultrafast bootstrap values (BS) ≥ 95% were interpreted as strong statistical support. All alignment matrices and tree files generated in this study have been archived in Zenodo (https://doi.org/10.5281/zenodo.19005486).

**Table 1.** Voucher information for specimens included in phylogenetic analyses in this study. Bold: sequences obtained in this study, *: type specimen.

Species	Voucher no.	Locality	Accession	
			ITS	LSU
*Agaricales* sp.	Sw5-1	Japan	AB859205	N/A
*Agaricales* sp.	Sw2-1	Japan	AB859204	N/A
*Connopus acervatus*	TENN-F-062879	Sweden	GU318387	FJ750255
*C. acervatus*	TENN-F-062824	USA	GU318393	FJ750260
*C. acervatus*	TENN-F-062825	USA	GU318395	FJ750259
*C. acervatus*	TENN-F-061292	Canada	GU318383	FJ750254
*Gymnopus adventitius* nom. prov.	SFSU: DED8813	N/A	KY026760	KY026760
*G. aff. dryophilus*	TENN-F-065157	Belgium	KY026679	KY026679
*G. aff. dryophilus*	TENN-F-065157	Belgium	KY026680	KY026680
*G. ailaoensis*	HKAS 131326*	China	OR815363	OR815381
*G. ailaoensis*	HKAS 131325	China	OR815364	OR815382
*G. ailaoensis*	HKAS 131324	China	OR815365	OR815383
*G. alkalivirens*	TENN-F-058672	Greenland	DQ480112	N/A
*G. alliifoetidissimus*	GDGM 76695*	China	MT023348	MT017526
*G. alpinus*	TENN-F-055834	Scotland	DQ480114	N/A
*G. androsaceus*	TFB5037	Canada	KY026749	N/A
*G. androsaceus*	RA725-21	USA	MN148655	N/A
*G. androsaceus*	TENN-F-059594	Russia	KY026663	KY026663
*G. androsaceus*	TFB 5021	Canada	KY026747	KY026747
*G. androsaceus*	TFB 5609	USA	KY026750	KY026750
*G. androsaceus*	TENN-F-069268 h2	Slovakia	KY696772	KY696772
*G. aquosus*	BRNM 665362	Czech Republic	JX536172	N/A
*G. aurantiipes*	SFSU: AWW118	Indonesia	AY263432	AY639410
*G. aurantiofuscus*	HGASMF01-7024*	China	PP151504	PP151551
*G. austrosemihirtipes*	AWW65*	Indonesia	AY263422	N/A
*G. barbipes*	TENN-F-067858*	USA	KJ416269	KY019642
*G. bicolor*	SFSU: AWW116*	Indonesia	AY263423	AY639411
*G. bicolor*	KUN-HKAS144469	China	PQ771157	PQ771142
*G. bisporus*	BCN:SCM B-4065*	Spain	JN247551	JN247555
*G. brassicolens*	TENN: F-059294	Austria	DQ449991	N/A
*G. brunneiniger*	XAL: Cesar49*	Mexico	MT232389	MW187070
*G. brunneostipitatus*	HMJAU 60412*	China	PP151535	PP639544
*G. bunerensis*	LAH 35878*	Pakistan	MK122772	MK122770
*G. catalonicus*	BCN:SCM B-4057*	Spain	JN247552	JN247556
*G. ceraceicola*	PDD:87181*	New Zealand	KC248405	N/A
*G. chowii*	HMJAU 60415*	China	PP151495	PP151548
*G. conifericola*	HMJAU 60413	China	PP151542	PP151571
*G. cremeostipitatus*	BRNM: 747547*	South Korea	KF251071	KF251091
*G. densilamellatus*	BRNM: 714927	South Korea	KP336685	KP336694
*G. dryophilus*	TENN-F-057012	N/A	DQ241781	AY640619
*G. dryophilus*	BRNM: 732938	Czech Republic	JX536149	N/A
*G. dysodes*	TENN-F-061125	USA	KY026666	FJ750265
*G. earleae*	TENN-F-059457	USA	AY256694	N/A
*G. efibulatus*	HGASMF01-7052*	China	OM970865	OM970865
*G. efibulatus*	HGASMF01-7052*	China	OM970866	OM970866
*G. efibulatus*	HGASMF01-11995	China	OM970873	N/A
*G. erythropus*	BRNM 664995	Czech Republic	JX536133	N/A
*G. erythropus*	BRNM 693553	Switzerland	JX536135	N/A
*G. flavoalbus*	KUN-HKAS144470*	China	PQ771152	PQ771137
*G. flavoalbus*	KUN-HKAS144471	China	PQ771153	PQ771138
*G. foetidus*	TENN-F-069280	Slovakia	KY026730	KY026730
*G. foetidus*	TENN-F-069323	USA	KY026739	KY026739
*G. frigidomarginatus* nom. prov.	TENN-F-055679	USA	KY026648	KY026648
***G. fuscostipes***	**HMJAU 60453c1**	**China**	**PP477385**	**N/A**
***G. fuscostipes***	**HMJAU 60453c2**	**China**	**PP477386**	**N/A**
***G. fuscostipes***	**HMJAU 60453c3**	**China**	**PP477387**	**N/A**
***G. fuscostipes***	**HMJAU 60453c4**	**China**	**PP477388**	**N/A**
***G. fuscostipes***	**HMJAU 60453c5**	**China**	**PP477389**	**N/A**
***G. fuscostipes***	HMJAU 60451*	**China**	**PP477384**	**PP477390**
***G. fuscostipes***	**HMJAU 60452**	**China**	**PP477383**	**N/A**
*G. fuscopurpureus*	BRNM:809119	Czech Republic	MZ542559	MZ542563
*G. fuscus*	GDGM 28929*	China	PP151515	N/A
*G. fusipes*	TENN-F-059217	France	AY256710	AY256710
*G. fusipes*	TENN-F-069254	Slovakia	KY026727	KY026727
*G. gansunensis*	MHGAU FLF541*	China	PP911508	PP907040
*G. gaoligongensis*	HKAS 145616*	China	PQ900212	PQ896818
*G. graveolens*	FF17084	France	MH422573	MH422572
*G. hakaroa*	PDD:87315*	New Zealand	KC248410	N/A
*G. imbricatus*	PDD:95489*	New Zealand	KC248390	N/A
*G. impudicus*	BRNM: 714849	Czech Republic	LT594119	LT594119
*G. impudicus*	JVG 1130531-2	Spain	LT594120	LT594120
*G. indoctoides*	SFSU: AWW125*	Indonesia	AY263424	AY639419
*G. inflatotrama* nom. prov.	TENN-F-048143	USA	KY026619	KY026619
*G. inflatotrama* nom. prov.	TENN-F-051233	USA	KY026632	KY026632
*G. inflatotrama* nom. prov.	TENN-F-053490	USA	KY026640	KY026640
*G. inflatotrama* nom. prov.	TFB 4529	USA	KY026744	KY026744
*G. inusitatus*	BCN:SCM B-4058*	Spain	JN247553	JN247557
G. inusitatus var. cystidiatus	BRNM: 737257*	Hungary	JN247550	MK278109
*G. iocephalus*	TENN-F-052970	USA	DQ449984	KY019630
*G. iodes*	HGASMF01-10068*	China	OM970869	OM970869
*G. irresolutus*	SFSU:DED 8209*	Sao Tome	MF100973	N/A
*G. jilongensis*	HMAS 300549*	China	PQ099856	PP968805
*G. linzhiensis*	HMAS 288012*	China	PV815626	PV815628
*G. montagnei*	JMCR 143	N/A	DQ449988	AF261327
*G. neobrevipes*	TENN-F-069197*	USA	MH673477	MH673477
*G. nigrescens*	AH:49188*	Spain	MZ542560	MZ542564
*G. niveus*	GDGM 70487*	China	PP151516	PP151560
*G. novae-angliae* nom. prov.	TFB 4975	USA	KY026745	KY026745
*G. novomundi* nom. prov.	SFSU: DED5097	USA	KY026759	KY026759
*G. ocior*	TENN-F-065135	Belgium	KY026678	KY026678
*G. ocior*	BRNM 737693	Norway	JX536164	N/A
*G. ocior*	KUN-HKAS83528	China	PQ771161	PQ771146
*G. omphalinoides*	GDGM 78318*	China	MW134044	MW134730
*G. omphalinoides*	GDGM 44411	China	MW134040	MW134726
*G. omphalinoides*	GDGM 78483	China	MW134045	MW134731
*G. omphalinoides*	HMJU 00506	China	MW134047	MW134733
*G. omphalinoides*	HKAS 107312	China	OK087326	N/A
*G. omphalinoides*	SL2111	Singapore	OR527364	N/A
*G. otagensis*	PDD:106823	New Zealand	MT974597	MT974601
*G. otagensis*	PDD:113265	New Zealand	MT974600	MT974602
*G. pallipes*	GDGM 81513*	China	MW582856	OK087327
*G. portoricensis*	TENN-F-051029*	Puerto Rico	KY026628	KY026628
*G. portoricensis*	TENN-F-051029*	Puerto Rico	KY026629	KY026629
*G. pseudoandrosaceus*	HGASMF01-23515*	China	PP939647	N/A
*G. pseudoandrosaceus*	HGASMF01-23386	China	PP935805	PQ724923
*G. pubipes*	AH:26931	Spain	MZ542558	MZ542562
*G. pygmaeus*	URM 90003*	Brazil	KX869966	KY088273
*G. salakensis*	AWW29	Indonesia	AY263447	N/A
*G. schizophyllus*	GDGM 77165*	China	MW134043	MW134729
*G. schizophyllus*	GDGM 77038	China	MW134042	MW134728
*G. schizophyllus*	GDGM 76287	China	MW134041	MW134727
*G. schizophyllus*	HKAS 96494	China	MW134046	MW134732
*G. semihirtipes*	TENN-F-007595	USA	OK376741	N/A
*G. sepiiconicus*	SFSU: AWW126	Indonesia	AY263449	AY639427
*G. similis*	BRNM: 766739*	South Korea	KP336692	KP336699
*G. similis*	BRNM 718713	South Korea	KP336691	KP336698
*G. similis*	BRNM 714981	South Korea	KP336690	KP336697
*G. sinobrevipes*	KUN-HKAS108042*	China	PQ771149	PQ771134
*G. sinobrevipes*	KUN-HKAS107802	China	PQ771151	PQ771136
*G. sinobrevipes*	KUN-HKAS108101	China	PQ771150	PQ771135
*G. sinopolyphyllus*	HMJAU 60386*	China	OM970872	OM970872
*G.* sp.	BRNM 718701	South Korea	MH589968	MH589987
*G.* sp.	TENN-F-055023	Argentina	KY559328	N/A
*G.* sp.	LE-BIN 5068	Viet Nam	OR883538	N/A
*G.* sp.	R061675	China	GU256981	N/A
*G.* sp.	Y-BL41	Japan	LC505333	N/A
*G.* sp.	Ta-BL62	Japan	LC505290	N/A
*G.* sp.	LE-BIN 5118	Viet Nam	OR883529	N/A
*G.* sp. 1	PUL:HONDURAS19-F001	Honduras	MT571521	N/A
*G.* sp. 5	TENN-F-66344	USA	KY026691	KY026691
*G. spongiosus*	TENN-F-065912	USA	KY026686	KY026686
*G. spongiosus*	TENN-F-068184	USA	KY026706	KY026706
*G. stipitovirens*	HMJAU 60420*	China	PP151528	N/A
*G. strigosipes*	HMAS 295796	China	OM970874	OM970874
*G. subsepiiconicus*	MHGAU FLF393*	China	PP911505	PP907039
*G. subsupinus*	PDD:96595	New Zealand	KM975399	KM975375
*G. talisiae*	URM 87730*	Brazil	KT222655	N/A
*G. tianbaoyanensis*	HMJAU 60417*	China	PP151500	N/A
*G. trabzonensis*	KATO:Fungi:3375*	Turkey	KT271754	N/A
*G. variicolor*	BRNM: 714959	South Korea	LT594121	KP348011
*G. variicolor*	BRNM 781307*	South Korea	KX926134	N/A
*G. variicolor*	BRNM 781305	South Korea	KX926133	N/A
*G. violaceigregarius*	LAH37854*	Pakistan	OR063896	OR063898
*G. viridocephalus*	HGASMF01-7003*	China	PP151513	PP151559
*G. viridocephalus*	KUN-HKAS132571	China	PQ771162	PQ771147
*G. viridocephalus*	KUN-HKAS132589	China	PQ771163	PQ771148
*G. vitellinipes*	SFSU: AWW127*	Indonesia	AY263429	AY639432
*G. westii*	FLAS-F-62667	USA	MK268237	N/A
*G. westii*	FLAS-F-62668	USA	MK268238	N/A
*G. wutaishanensis*	BJTC FM484*	China	OK661075	OK663007
*G. yunnanensis*	KUN-HKAS108570*	China	PQ771154	PQ771139
*G. yunnanensis*	KUN-HKAS108610	China	PQ771155	PQ771140
*G. yunnanensis*	KUN-HKAS144472	China	PQ771156	PQ771141
*Marasmiellus praeacutus*	iNAT:236189295	USA	PX404267	N/A
*Marasmius* sp.	Ej4-1	Japan	LC504889	N/A
*Ma.* sp.	KA12-0382	South Korea	KR673444	N/A
*Micromphale arbuticola*	FDS-CA-03602	USA	PQ144490	N/A
*Mi. foetidum*	NEHU.MBSRJ.48	India	KP877447	N/A
*Paragymnopus magnisporus*	HMJAU 60432*	China	PP151534	PP151567
*Pg. perforans*	TENN-F-050318	Sweden	KY026623	KY026623
***Pseudomarasmius brunneodiscus***	HMJAU 60404*c2	**China**	**OQ586185**	**OQ586185**
***Ps. brunneodiscus***	HMJAU 60404*c5	**China**	**OQ586186**	**OQ586186**
***Ps. brunneodiscus***	**HMJAU 60455**	**China**	**PP477379**	**N/A**
***Ps. brunneodiscus***	**HMJAU 60409**	**China**	**PP477380**	**N/A**
***Ps. brunneodiscus***	**HMJAU 60454**	**China**	**PP477381**	**N/A**
***Ps. brunneodiscus***	**HMJAU 60456**	**China**	**PP477382**	**N/A**
*Ps. efibulatus*	TENN-F-056187*	New Zealand	MK268234	N/A
*Ps. glabrocystidiatus*	BRNM: 718676*	South Korea	KF251073	KF251092
*Ps. glabrocystidiatus*	HMJAU 60437	China	PP151525	PP151564
*Ps. glabrocystidiatus*	HKAS 133552	China	PV715544	N/A
*Ps. nidus-avis*	TENN-F-069310	USA	KY026732	KY026732
*Ps. nidus-avis*	XAL: Cesar36*	Mexico	MH560576	N/A
*Ps. pallidocephalus*	TENN-F-052401	USA	KY026635	KY026635
*Ps. pallidocephalus*	TENN-F-065829	USA	KY026684	KY026684
*Ps. pallidocephalus*	TENN-F-065829	USA	KY026685	KY026685
*Ps. pallidocephalus*	SL228	N/A	MW816527	N/A
*Ps. pallidocephalus*	20211415Marasmius	China	OR198338	N/A
*Ps. pallidocephalus*	LE-BIN 5132	USA	OR936458	N/A
*Ps. pallidocephalus*	LE-BIN 5162	Viet Nam	OR936459	N/A
*Ps. pallidocephalus*	HAY-F-003748	USA	PQ644464	N/A
*Ps. patagonianus*	TENN-F-054424*	KY352649	KY352649	N/A
*Ps. quercophylloides*	TENN-F-049177*	China	MK268235	N/A
*Ps. quercophylloides*	GDGM 60244	China	PP151510	PP151556

Dataset 1 (ITS–LSU) included 151 sequences aligned across 1812 columns, of which 443 were parsimony-informative, 136 were singleton, and 1233 were constant. The alignment was partitioned into four regions corresponding to ITS1 (positions 1–351), 5.8S (352–511), ITS2 (512–984), and LSU (985–1812) to evaluate best-fit partitioning schemes and substitution models. In the maximum likelihood analysis, a two-partition scheme was selected: ITS1 and ITS2 were merged and assigned the TIM2+F+I+G4 model, while 5.8S and LSU were merged under the HKY+F+I+G4 model. In the Bayesian analysis, a corresponding two-partition scheme was implemented. Both the combined ITS1/ITS2 partition and the combined 5.8S/LSU partition were modeled under GTR+I+G4. The MCMC analysis was automatically terminated at 2,775,000 generations when the ASDSF fell below 0.01. LnL trace plots showed no obvious trend after burn-in.

Dataset 2 (ITS–LSU) comprised 32 sequences and 1656 aligned sites, with 188 parsimony-informative, 31 singleton, and 1437 constant sites. The alignment was initially divided into four regions corresponding to ITS1 (positions 1–323), 5.8S (324–483), ITS2 (484–787), and LSU (788–1656) to evaluate best-fit partitioning schemes and substitution models. In the maximum likelihood analysis, a two-partition scheme was selected: ITS1 and ITS2 were merged and assigned the HKY+F+G4 model, while 5.8S and LSU were merged under the TN+F+I model. In the Bayesian analysis, ITS1 and ITS2 were merged into a single partition modeled under HKY+F+G4. The 5.8S and LSU regions were treated as separate partitions. For the 5.8S partition, although model selection suggested SYM+I, we employed the GTR+I model with estimated base frequencies (consistent with the parameterization for LSU) to avoid the strict assumption of equal state frequencies. The MCMC analysis stopped automatically at 785,000 generations when the average standard deviation of split frequencies dropped below 0.01. LnL trace plots showed no obvious trend after burn-in.

## Results

### Phylogeny

**Figure 1.**
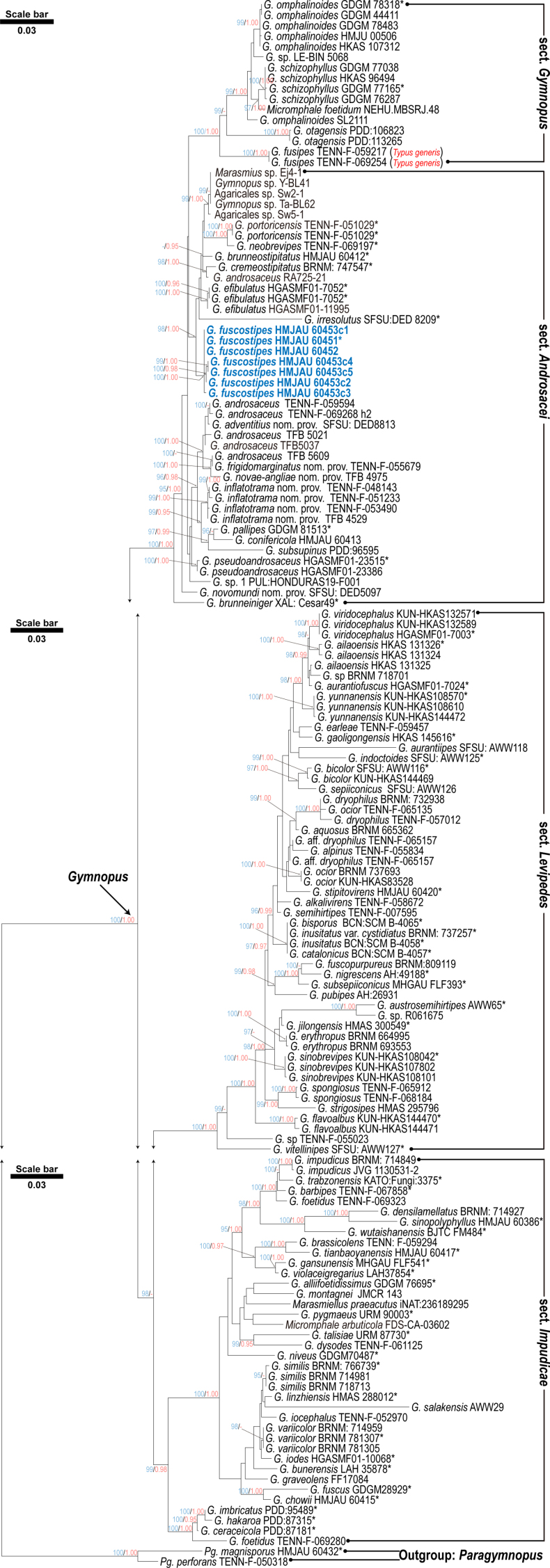
Phylogenetic tree of *Gymnopus* reconstructed from the combined ITS and LSU dataset based on the ML method. *Paragymnopus magnisporus* and *Pg. perforans* were selected as the outgroups. BS values ≥ 95% and PP values ≥ 0.95 are shown above the nodes. The new taxon was highlighted in bold.

The tree for Dataset 1 (Fig. 1) shows that the sampled *Gymnopus* taxa formed a well-supported clade (BS = 100%, PP = 1.0). All infrageneric sections formed well-supported monophyletic clades, whereas *sect. Androsacei* remained unresolved. The new materials (HMJAU 60451–60453) clustered together in a new independent, strongly supported lineage (BS = 100%, PP = 1.0). This lineage was nested within a larger clade (BS = 98%, PP = 1.0) that includes several species currently assigned to *sect. Androsacei*. This clade comprises species such as *G. brunneostipitatus* J.P. Li, Chang-Tian Li & Y. Li, *G. cremeostipitatus* Antonín, Ryoo & Ka, *G. efibulatus* J.P. Li, Chang-Tian Li, Chun Y. Deng & Y. Li, *G. irresolutus* Desjardin & B.A. Perry, *G. neobrevipes* R.H. Petersen, and *G. portoricensis* R.H. Petersen, indicating a close phylogenetic relationship among these taxa. In addition to these well-identified species, the clade also contained several unidentified sequences (Ej4-1, Y-BL41, Sw2-1, Ta-BL62, Sw5-1). One sequence labeled *G. androsaceus* (RA725-21) was also placed in this clade, whereas the majority of *G. androsaceus* sequences formed a separate lineage elsewhere in the tree, indicating that the identification of this accession requires further verification.

**Figure 2.**
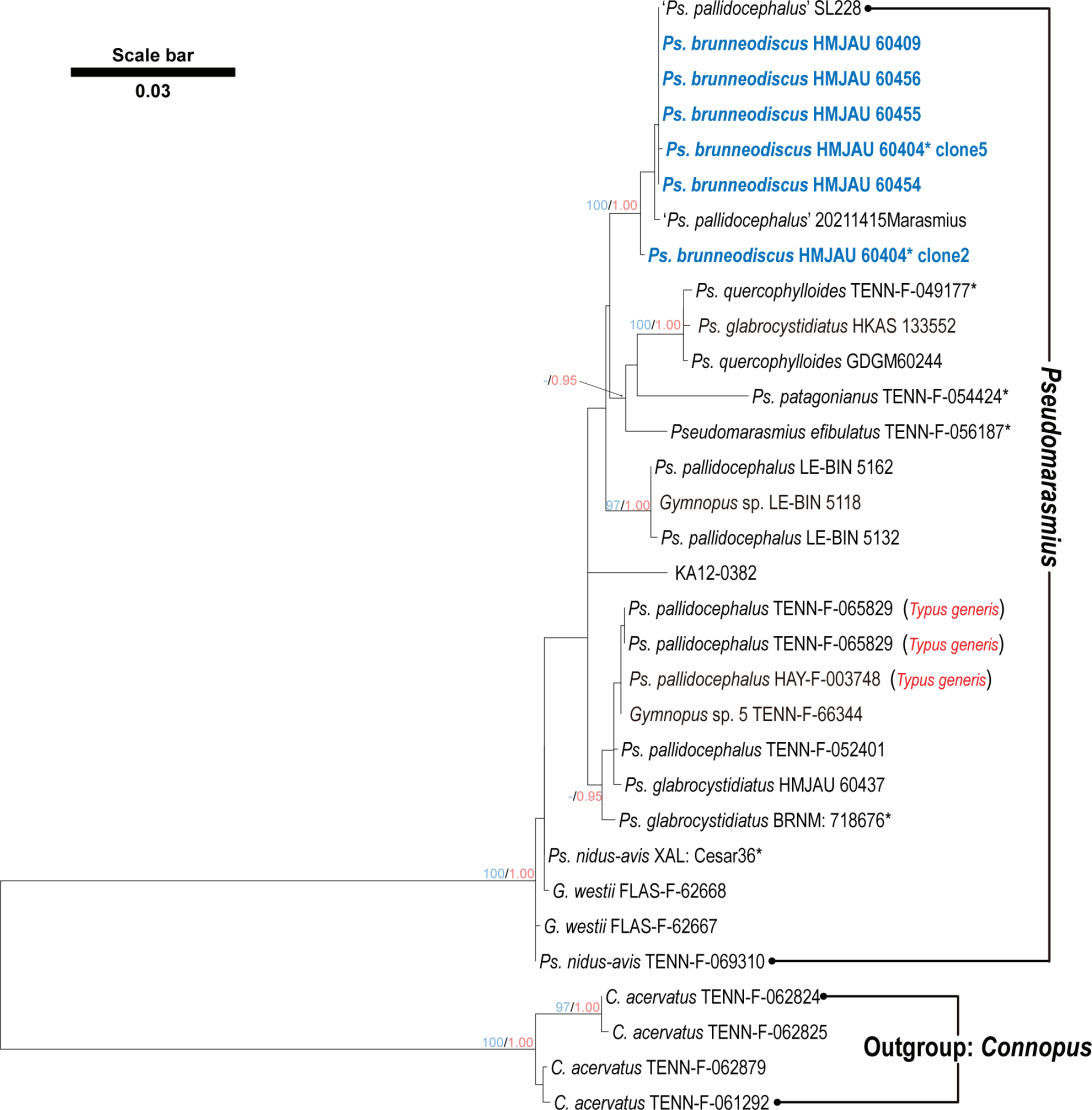
Phylogenetic tree of *Pseudomarasmius* reconstructed from the combined ITS and LSU dataset based on the ML method. *Connopus acervatus* was selected as the outgroup. BS values ≥ 95% and PP values ≥ 0.95 are shown above the nodes. The new taxon is in bold.

The tree of Dataset 2 (Fig. 2) shows *Pseudomarasmius* formed a robust clade (BS = 100%, PP = 1.0). The new samples (HMJAU 60404, HMJAU 60409, HMJAU 60454–60456), together with two sequences labeled *Ps. pallidocephalus* (SL228, 20211415Marasmius), cluster in a separate, strongly supported lineage (BS = 100%, PP = 1.0), indicating that they are conspecific. However, two additional, distantly related lineages in the tree also bear the name *Ps. pallidocephalus*, one comprising three sequences (LE-BIN 5162, LE-BIN 5118, and LE-BIN 5132) and the other including six samples (TENN-F-065829, HAY-F-003748, TENN-F-66344, TENN-F-052401, HMJAU 60437, and BRNM: 718676*). This pattern indicates that the status of these collections in relation to *Ps. pallidocephalus* requires further clarification based on detailed morphological comparisons and molecular data from reliably identified material.

### Taxonomy

#### Gymnopus fuscostipes

Taxon classificationFungiAgaricalesOmphalotaceae

J.P. Li & Chang-Tian Li sp. nov.

CB3EDAE7-2FEF-52C6-BC72-E5DF110966B6

Fungal Names: FN 571628

Figs 3, 4

##### Holotype.

China • Yunnan Province, Diqing Tibetan Autonomous Prefecture, Shangri-La City; 28°14'51"N, 99°58'28"E; elev., 3917 m; 01 August 2022; M.C. Pan; PMC441 (HMJAU 60451).

**Figure 3.**
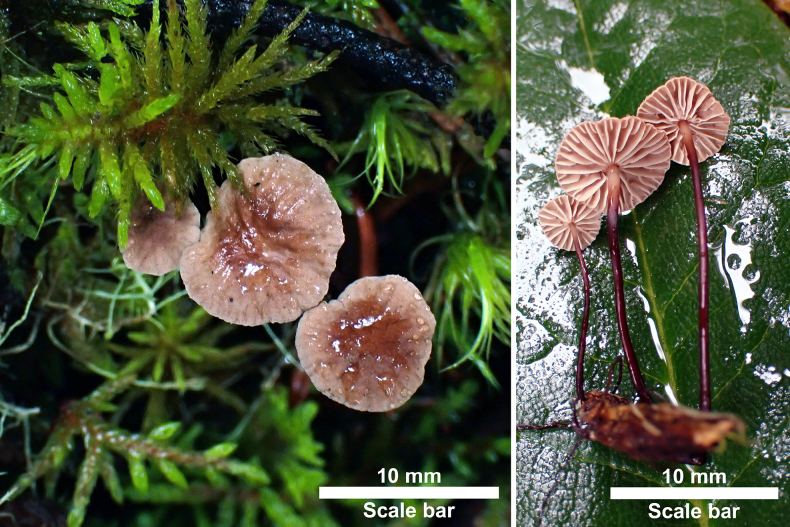
Basidiomata of *Gymnopus fuscostipes*. HMJAU 60451 (PMC441, holotype). Photos by: M.C. Pan.

**Figure 4.**
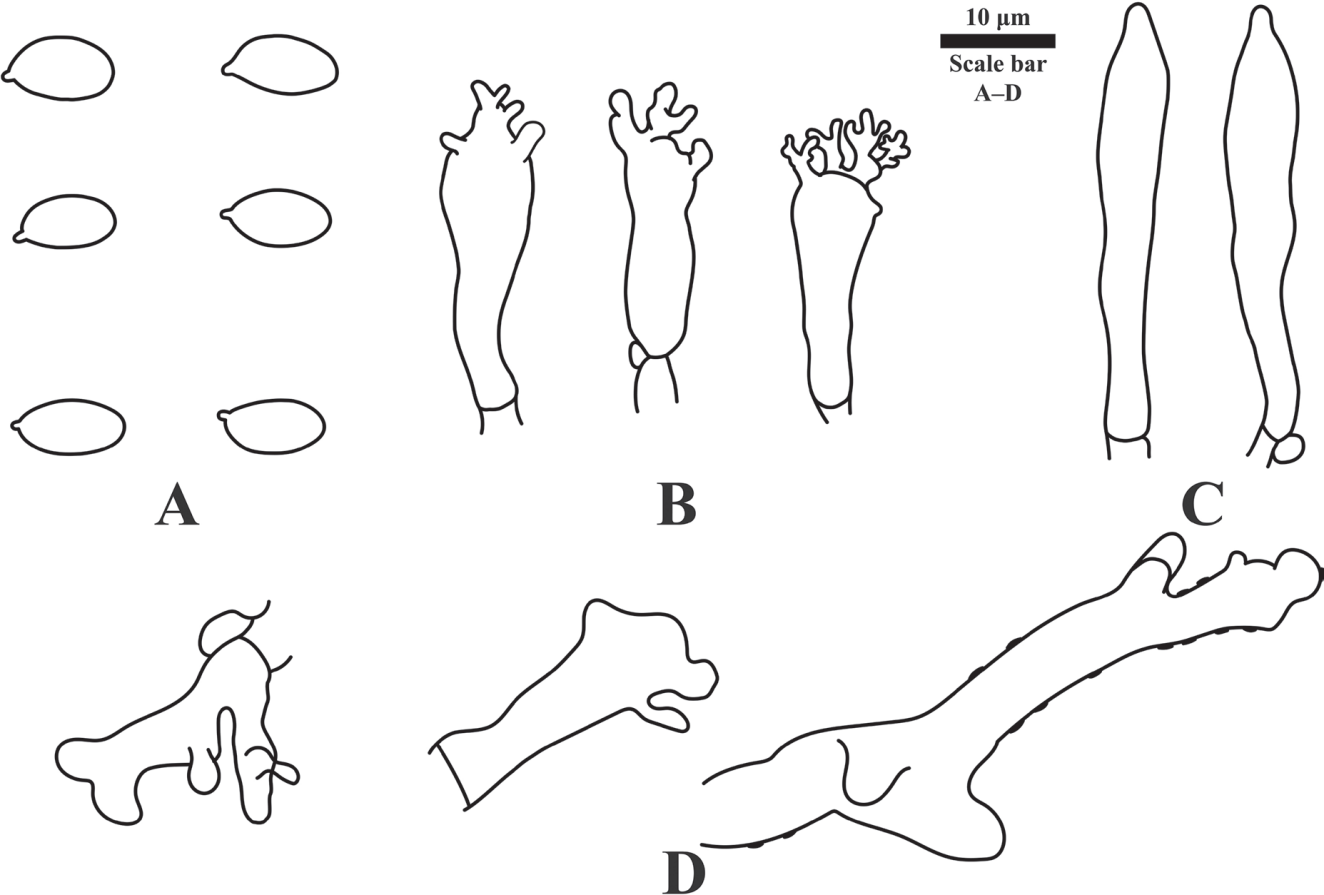
Microscopic features of *Gymnopus fuscostipes*. HMJAU 60451 (PMC441, holotype). **A**. Basidiospores; **B**. Cheilocystidia; **C**. Pleurocystidia; **D**. Pileipellis terminal cells. Scale bars: 10 μm. Drawings by: J.P. Li.

##### Etymology.

Derived from Latin, ‘*fuscostipes*’ merges ‘*fuscus*’ (dark brown to nearly black) with ‘*stipes*’ (stipe), denoting the dark brown to nearly black stipe.

##### Diagnosis.

Similar to *G. androsaceus*, but differs by its relatively large basidiospores (8.5–9.8 × 4.6–5.4 μm) and the absence of typical broom cells in the pileipellis.

##### Description.

***Basidiomata*** marasmioid. ***Pileus*** 5–12 mm diam, membranous, hemispherical to convex when young, becoming plano-convex to applanate with age, often depressed at the disc, radially sulcate towards margin, often rugose somewhere, dry, dark brown (9F9) overall when young, then reddish brown to dark brown (8D8–8F8) at disc, gradually fading to reddish white (7A2) towards margin zone, nearly white at margin, mostly orange grey (6B2) overall when mature. ***Lamellae*** subdistant, L = 10–17, l = 2–4, up to 1.5 mm broad, adnate, often ventricose, sometimes attached to a pseudocollarium, pinkish (7A2), reddish grey (7B2). ***Stipe*** 17–40 mm long, up to 1 mm thick at middle, centrally attached and insititious, broadened at apex or base, often cylindrical, sometimes compressed towards base, chitinous, dry, glabrous, hollow, almost dark brown (8F8 to 9F8) overall, fading to reddish white (7A2) at apical zone, black overall when mature. ***Rhizomorphs*** present, concolorous with stipe, shiny, wiry, simple, repent. ***Odor*** negligible.

***Basidiospores*** [*n* = 20, *m* = 2] 8.5–9.8(–9.9) × 4.6–5.4(–5.6) μm (average = 9.13 ± 0.41 × 4.93 ± 0.30 μm, Q = (1.68–)1.71–1.97(–2.00), Qm = 1.86 ± 0.09), oblong. ***Basidia*** [*n* = 10] 25.1–38.2 × 7.1–9.2 μm, 4-spored, clavate, cylindrical. ***Basidioles*** [*n* = 10, *m* = 2] 22.7–38.4 × 6.5–8.7 μm, clavate, cylindrical. ***Cheilocystidia*** [*n* = 10, *m* = 2] 13.3–28.2 × 4.3–7.9 μm, fusiform to clavate or irregularly, with fine apical branches or not, often mostly as ***Siccus***-type broom cells, sometimes only with less finger-like apical projections. ***Pleurocystidia*** [*n* = 10, *m* = 2] 27.9–42.3 × 5.8–6.8 μm, fusiform. ***Pileipellis*** a cutis composed of cylindrical hyphae, thin-walled to slightly thick-walled, incrusted, hyaline; terminal cells often more or less diverticulate, lobed to irregularly branched, sometimes cylindrical, mixed with some having coralloid structure, broom cells not found. ***Stipitipellis*** a cutis composed of cylindrical, thin-walled, hyaline, parallel-arranged hyphae, dextrinoid, smooth. ***Caulocystidia*** absent. ***Clamp connections*** present in all tissues.

##### Ecology.

Saprotrophic, solitary to scattered, usually arising on fallen broad leaves in mixed broadleaf and coniferous forests.

##### Additional specimens examined.

China • Yunnan Province, Diqing Tibetan Autonomous Prefecture, Shangri-La City; 28°14'39"N, 99°58'26"E; elev., 3873 m; 01 August 2022; J.P. Li; LJP1474 (HMJAU 60452). • Yunnan Province, Diqing Tibetan Autonomous Prefecture, Shangri-La City; 28°19'53"N, 99°07'19"E; elev., 4164 m; 03 August 2022; J.P. Li; LJP1515 (HMJAU 60453).

#### Pseudomarasmius brunneodiscus

Taxon classificationFungiAgaricalesOmphalotaceae

J.P. Li, Chang-Tian Li & Yi Li sp. nov.

7C9CC42D-62AB-5090-96F0-38C28D304265

Fungal Names: FN 571335

Figs 5, 6

##### Holotype.

China • Zhejiang Province, Zhuji City, Chenzhai Town, Wu’er Village; 29°27'33"N, 120°21'13"E; elev. 198 m; 14 September 2022; Yi Li & M.L. Xie; Liyi4992 (HMJAU 60404).

**Figure 5.**
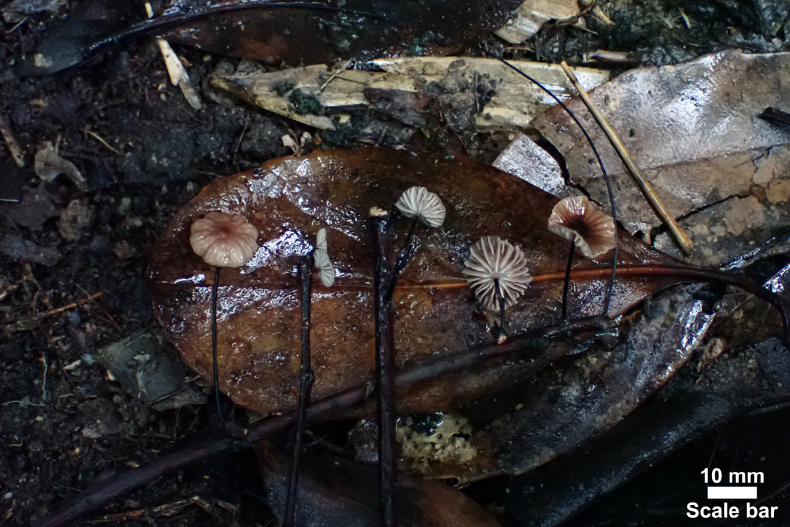
Basidiomata of *Pseudomarasmius brunneodiscus*. HMJAU 60404 (holotype). Photo by: M.L. Xie.

**Figure 6.**
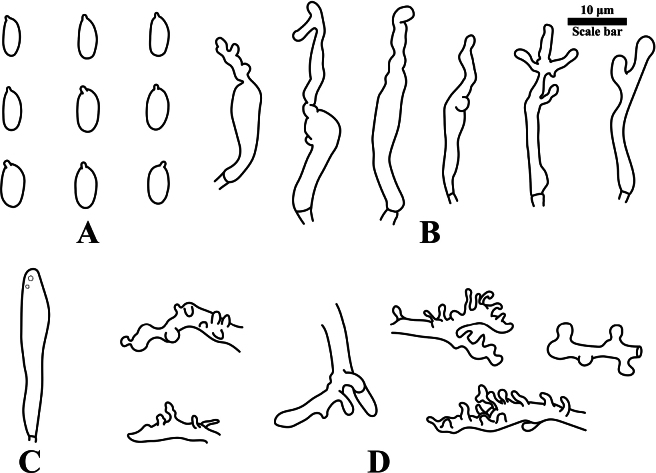
Microscopic features of *Pseudomarasmius brunneodiscus*. HMJAU 60404 (holotype). **A**. Basidiospores; **B**. Cheilocystidia; **C**. Pleurocystidia; **D**. Pileipellis cells. Scale bars: 10 μm. Drawings by: J.P. Li.

##### Etymology.

The specific name “*brunneodiscus*” (Lat.) refers to the brown pileus disc of the new species.

##### Diagnosis.

Similar to *Ps. glabrocystidiatus*, but different in its occurrence in broadleaf forests, smaller basidiospores (7.16 × 3.32 μm), and its distinct phylogenetic placement.

##### Description.

***Basidiomata*** marasmioid. arising from woody substrate or as side branches of rhizomorphs. ***Pileus*** 5–15 mm broad, membranous, plane-convex to applanate, depressed or umbonate at disc, sulcate towards margin, sometimes deflexed or reflexed from the sides of the margin towards the center, margin slightly crenate, dull red (8B3) to dark brown (8F8) at disc, more or less paler towards margin. ***Lamellae*** distant, L = 10–16, l = 1–2, adnate to subdecurrent, seceding to appear pseudocollariate, white or tincted with color of pileus. ***Stipe***12–42 mm long, less than 1 mm wide, cylindrical, bristle-like, glabrous, insititious, central, black overall. ***Rhizomorphs*** present, (> 400 × 0.03–0.46 mm), black, shiny, wiry, simple, arising from the substrate.

***Basidiospores*** [*n* = 40, *m* = 4] (6–)6.5–8 × 3–4 μm (average = 7.16 ± 0.49 × 3.32 ± 0.28 μm, Q = (1.81–)1.84–2.51(–2.65), Qm = 2.17 ± 0.21), oblong to cylindric. ***Basidia*** [*n* = 20] 19–34 × 5–7 μm, 4-spored, clavate, clamped but rare. ***Basidioles*** [*n* = 20, *m* = 2] 21–31 × 5–7 μm, clavate, clamped but rare. ***Cheilocystidia*** [*n* = 30, *m* = 3] 15.5–34.5 × 2.5–6 μm, more or less irregularly cylindrical to clavate, with irregular filiform apical projection, sometimes branched at upper part, clamp connections not observed. ***Pleurocystidia*** [*n* = 20, *m* = 2] 17–33 × 3.5–5.5 μm, obscure, fusiform, clamped but rare. ***Pileipellis*** a cutis, embedded in a thin slime matrix, composed of cylindrical hyphae, which are thin-walled, incrusted or not, knobbed, diverticulate to forming a demonstrable ***ramealis***-structure, hyaline, clamp connections not observed. ***Stipitipellis*** a cutis composed of cylindrical, thick-walled, hyaline, parallel-arranged hyphae, smooth. ***Caulocystidia*** absent.

##### Ecology.

Saprotrophic, gregarious, arising on dead twigs, branches, or rhizomorphs in broadleaf forests.

##### Additional specimens examined.

China • Chongqing City, Banan District; 29°42'25"N, 106°58'24"E; latitude, longitude, and elevation not recorded; 25 September 2023; X. Li; LX20230925–004 (HMJAU 60409). • Zhejiang Province, Jinhua City, Wuyi County; latitude, longitude, and elevation not recorded; 06 October 2023; C.M. Chen; CCM001 (HMJAU 60454). • Fujian Province, Yongan City, Tianbaoyan National Nature Reserve; latitude and longitude not recorded; elev., 680 m; 01 August 2023; J.P. Liao & H.X. Luo; TBY20230801–001 (HMJAU 60455). • Fujian Province, Yongan City, Tianbaoyan National Nature Reserve; latitude and longitude not recorded; elev., 680 m; 22 August 2023; J.P. Liao & H.X. Luo; TBYLJP20230822–008 (HMJAU 60456).

## Discussion

*Gymnopus fuscostipes* is a marasmioid species characterized by dark-colored stipes and well-developed rhizomorphs. In comparison with morphologically similar species, *G. neobrevipes* is distinguished by its rather distant lamellae, the rather short stipe (≤ 6 mm), and smaller basidiospores (mean length = 8.1 µm, Petersen and Hughes 2019), whereas *G. portoricensis* is characterized by distant lamellae (L = 7–10), a very small stipe (1–2.5 mm), and smaller basidiospores (mean length = 6.58 µm, Petersen and Hughes 2019), and *G. androsaceus* differs in relatively small basidiospores (7.0–9.0 × 3.5–4.5 μm) and the presence of broom cells in the pileipellis (Antonín and Noordeloos 2010). Among its closely related species within the phylogenetic tree (Fig. 1), *G. brunneostipitatus* exhibits a distinct morphology with smaller basidiospores (mean size = 7.9 × 4.2 μm) and the presence of caulocystidia (Li et al. 2024); *G. cremeostipitatus* is characterized by its cream stipe, smaller basidiospores (mean size = 7.1 × 3 μm), and the presence of caulocystidia (Antonín et al. 2014); *G. efibulatus* stands out due to its whitish to yellowish rhizomorphs, smaller basidiospores (mean size = 7.8 × 4.3 μm), and the absence of clamp connections (Li et al. 2022); lastly, *G. irresolutus* can be identified by its pruinose stipe, smaller basidiospores (mean size = 7.8 × 3.5 μm), and the absence of clamp connections (Desjardin and Perry 2017). Furthermore, the phylogenetic relationship suggests that this species is closely related to *G. androsaceus*. However, the identification of the sequence labeled as *G. androsaceus* (RA725-21) requires further investigation, as it is distantly related to most sequences representing *G. androsaceus* (TENN-F-059594, TENN-F-069268h2, TFB 5021, TFB 5037, and TFB 5609) and falls into a different clade. Nevertheless, the morphological differences between *G. androsaceus* and the newly described species have already been discussed above.

*Pseudomarasmius brunneodiscus* is also a marasmioid species characterized by dark-colored stipes and well-developed rhizomorphs. Morphologically, the majority of species within *Pseudomarasmius* lack clamp connections, except for *Ps. nidus-avis* (E. César, Bandala and Montoya) R.H. Petersen and the new species (Petersen and Hughes 2020). However, for *Ps. nidus-avis*, the pileipellis is not embedded in a slime matrix, the clamp is only found in the stipe, and it is distributed in the North American Gulf Coast and subtropical Mexico (Petersen and Hughes 2020). *Pseudomarasmius efibulatus* R.H. Petersen is characterized by relatively distant lamellae (L = 7–8 through lamellae), shorter rhizomorphs (< 40 × 0.1–0.3 mm), larger basidiospores (mean length = 8.67 µm), and arising from needles of *Dacrydium* (Petersen and Hughes 2020). *Pseudomarasmius glabrocystidiatus* (Antonín, Ryoo & Ka) R.H. Petersen has larger basidiospores (8.8 × 4.4 μm), smooth cheilocystidia of broadly clavate or pyriform, and living on needles of *Abies holophylla* (Antonín et al. 2014). *Pseudomarasmius obscurus* R.H. Petersen is distinctive by having an estriate pileus, shorter rhizomorphs (≤ 25 mm), and variously shaped caulocystidia (Petersen and Hughes 2020). *Pseudomarasmius pallidocephalus* (Gilliam) R.H. Petersen differs from the new species by its habit of living in troops on needles and debris of conifers, shorter rhizomorphs (≤ 17 × 0.1–0.5 mm), and scattered cheilocystidia with the shape of clavate to ampulliform (Petersen and Hughes 2020). *Pseudomarasmius patagonianus* R.H. Petersen has larger basidiospores (9–9.5 × 4–5 µm), caulocystidia, and a pileipellis not embedded in a thin slime matrix (Petersen and Hughes 2020). *Pseudomarasmius quercophylloides* R.H. Petersen has a microscopically pruinose stipe minutely decorated with hyaline caulocystidia and narrower basidiospores (Q = 1.53, Petersen and Hughes 2020). *Pseudomarasmius straminipes* (Peck) R.H. Petersen has an off-white stipe, shorter rhizomorphs (< 20 × 0.1–0.3 mm), and is gregarious to scattered on dead conifer needles (Petersen and Hughes 2020).

*Gymnopus fuscostipes* and *Ps. brunneodiscus* fit well the concept of *G. sect. Androsacei* sensu Antonín and Noordeloos (2010), sharing marasmioid basidiomata, dark-colored stipes, and well-developed rhizomorphs. The main difference between the two species lies in the state of clamp connections: in *G. fuscostipes*, clamp connections are commonly present in all structures, whereas in *Ps. brunneodiscus*, the clamp only occasionally occurs in the basidia, basidiole, and pleurocystidia, and is not observed elsewhere. However, the presence or absence of clamp connections is not a consistent character in either group (see morphological comparisons above for exceptional species), which complicates the accurate delimitation of genera when relying only on morphological evidence. Given this instability, based on combined morphological and molecular phylogenetic evidence, *G. fuscostipes* and *Ps. brunneodiscus* are recognized as two new species, and their generic placement is confirmed.

## Supplementary Material

XML Treatment for Gymnopus fuscostipes

XML Treatment for Pseudomarasmius brunneodiscus

## Additional information

### Artificial Intelligence (AI) use

The authors accept full responsibility for the content of the manuscript, including the disclosure of any use of AI.

No AI tools were used in the preparation of this manuscript.

### Author ORCIDs

J.P. Li https://orcid.org/0000-0002-3250-7346

J. Gao https://orcid.org/0000-0002-8262-9069

Y. Li https://orcid.org/0000-0002-3482-5487

M.L. Xie https://orcid.org/0000-0002-7798-7048

C.T. Li https://orcid.org/0000-0001-6498-8642

### Data availability

All the sequences have been deposited in GenBank (https://www.ncbi.nlm.nih.gov) and Fungal Names (https://nmdc.cn/fungalnames/). All alignment matrices and tree files generated in this study have been archived in Zenodo (https://doi.org/10.5281/zenodo.19005486).
